# Elena+ Care for COVID-19, a Pandemic Lifestyle Care Intervention: Intervention Design and Study Protocol

**DOI:** 10.3389/fpubh.2021.625640

**Published:** 2021-10-21

**Authors:** Joseph Ollier, Simon Neff, Christine Dworschak, Arber Sejdiji, Prabhakaran Santhanam, Roman Keller, Grace Xiao, Alina Asisof, Dominik Rüegger, Caterina Bérubé, Lena Hilfiker Tomas, Joël Neff, Jiali Yao, Aishah Alattas, Veronica Varela-Mato, Amanda Pitkethly, Mª Dolores Vara, Rocío Herrero, Rosa Mª Baños, Carolina Parada, Rajashree Sundaram Agatheswaran, Victor Villalobos, Olivia Clare Keller, Wai Sze Chan, Varun Mishra, Nicholas Jacobson, Catherine Stanger, Xinming He, Viktor von Wyl, Steffi Weidt, Severin Haug, Michael Schaub, Birgit Kleim, Jürgen Barth, Claudia Witt, Urte Scholz, Elgar Fleisch, Florian von Wangenheim, Lorainne Tudor Car, Falk Müller-Riemenschneider, Sandra Hauser-Ulrich, Alejandra Núñez Asomoza, Alicia Salamanca-Sanabria, Jacqueline Louise Mair, Tobias Kowatsch

**Affiliations:** ^1^Centre for Digital Health Interventions, Department of Management, Technology and Economics, Eidgenössische Technische Hochschule (ETH) Zurich, Zurich, Switzerland; ^2^Department of Management, Technology, and Economics, Eidgenössische Technische Hochschule (ETH) Zurich, Zurich, Switzerland; ^3^Department of Psychology, University of Zurich, Zurich, Switzerland; ^4^Future Health Technologies, Singapore-ETH Centre, Campus for Research Excellence and Technological Enterprise (CREATE), Singapore, Singapore; ^5^School of Medicine, Johns Hopkins University, Baltimore, MD, United States; ^6^Executive School of Management, Technology and Law, University of St. Gallen, St. Gallen, Switzerland; ^7^School of Sport, Exercise and Health Sciences, Loughborough University, Loughborough, United Kingdom; ^8^Sport, Exercise and Health Sciences, Edinburgh Napier University, Edinburgh, United Kingdom; ^9^Polibienestar Research Institute, University of Valencia, Valencia, Spain; ^10^Centro de Investigación Biomédica en Red de Fisiopatología de la Obesidad y Nutrición (CIBERObn) Physiopathology of Obesity and Nutrition, Instituto de Salud Carlos III, Madrid, Spain; ^11^Department of Personality, Evaluation and Psychological Treatment, Faculty of Psychology, University of Valencia, Valencia, Spain; ^12^Department of Psychology, Universidad San Buenaventura, Bogotá, Colombia; ^13^National Institute of Education, Nanyang Technological University, Singapore, Singapore; ^14^Interdisciplinary Center for Health Workplaces, University of California, Berkeley, Berkeley, CA, United States; ^15^Centre for Digital Health Interventions, Institute of Technology Management, University of St. Gallen, St. Gallen, Switzerland; ^16^Department of Psychology, University of Hong Kong, Pokfulam, Hong Kong, SAR China; ^17^Department of Computer Science, Dartmouth College, Hanover, NH, United States; ^18^Center for Technology and Behavioral Health, Geisel School of Medicine, Hanover, NH, United States; ^19^Business School, Durham University, Durham, United Kingdom; ^20^Epidemiology, Biostatistics and Prevention Institute, University of Zurich, Zurich, Switzerland; ^21^Institute for Implementation Science in Health Care, University of Zurich, Zurich, Switzerland; ^22^Department of Psychiatry, Psychotherapy and Psychosomatics, University of Zurich, Zurich, Switzerland; ^23^Swiss Research Institute for Public Health and Addiction, University of Zurich, Zurich, Switzerland; ^24^Institute for Complementary and Integrative Medicine, University Hospital Zurich, University of Zurich, Zurich, Switzerland; ^25^Applied Social and Health Psychology, Department of Psychology, University of Zurich, Zurich, Switzerland; ^26^Dynamics of Healthy Aging, University of Zurich, Zurich, Switzerland; ^27^Family Medicine and Primary Care, Lee Kong Chian School of Medicine, Nanyang Technological University, Singapore, Singapore; ^28^Department of Medicine, Saw Swee Hock School of Public Health, Yong Loo Lin School of Medicine, National University of Singapore, Singapore, Singapore; ^29^Center for Digital Health, Berlin Institute of Health and Charité, Berlin, Germany; ^30^Department of Applied Psychology, University of Applied Sciences Zurich, Zurich, Switzerland; ^31^Unidad Académica de Cultura, Universidad Autónoma de Zacatecas, Zacatecas, Mexico

**Keywords:** chatbot, conversational agent (CA), digital coaching, digital health, coronavirus–COVID-19, gamification, mental health, pandemic lifestyle care

## Abstract

**Background:** The current COVID-19 coronavirus pandemic is an emergency on a global scale, with huge swathes of the population required to remain indoors for prolonged periods to tackle the virus. In this new context, individuals' health-promoting routines are under greater strain, contributing to poorer mental and physical health. Additionally, individuals are required to keep up to date with latest health guidelines about the virus, which may be confusing in an age of social-media disinformation and shifting guidelines. To tackle these factors, we developed Elena+, a smartphone-based and conversational agent (CA) delivered pandemic lifestyle care intervention.

**Methods:** Elena+ utilizes varied intervention components to deliver a psychoeducation-focused coaching program on the topics of: COVID-19 information, physical activity, mental health (anxiety, loneliness, mental resources), sleep and diet and nutrition. Over 43 subtopics, a CA guides individuals through content and tracks progress over time, such as changes in health outcome assessments per topic, alongside user-set behavioral intentions and user-reported actual behaviors. Ratings of the usage experience, social demographics and the user profile are also captured. Elena+ is available for public download on iOS and Android devices in English, European Spanish and Latin American Spanish with future languages and launch countries planned, and no limits on planned recruitment. Panel data methods will be used to track user progress over time in subsequent analyses. The Elena+ intervention is open-source under the Apache 2 license (MobileCoach software) and the Creative Commons 4.0 license CC BY-NC-SA (intervention logic and content), allowing future collaborations; such as cultural adaptions, integration of new sensor-related features or the development of new topics.

**Discussion:** Digital health applications offer a low-cost and scalable route to meet challenges to public health. As Elena+ was developed by an international and interdisciplinary team in a short time frame to meet the COVID-19 pandemic, empirical data are required to discern how effective such solutions can be in meeting real world, emergent health crises. Additionally, clustering Elena+ users based on characteristics and usage behaviors could help public health practitioners understand how population-level digital health interventions can reach at-risk and sub-populations.

## Introduction

The emergence of the COVID-19 coronavirus pandemic has created a global health emergency on an unprecedented scale (1). From the call to arms of research to tackle the pandemic, comes the challenge of delivering what the authors term *pandemic lifestyle care*. Pandemic lifestyle care concerns boosting population-level health during a period when many typical health promoting routines are severely disrupted and simple health promoting behaviors such as going for a walk or having personal space for relaxing hobbies have become much more difficult (2). Problems related to social isolation requirements have been rising; including flaunting of social distancing rules, lack of health promoting behaviors (physical activity, nutrition, sleep routines) and mental health issues (anxiety, loneliness) (3–5). In such circumstances, without additional intervention, individuals' health and well-being may deteriorate, particularly among at-risk groups with whom a lower level of health literacy, self-efficacy and/or access to resources exists already (6, 7). Additionally, without trusted resources and guidance readily and freely available at the population level, individuals may be more likely to ignore governmental guidelines, undermining public health efforts to tackle the pandemic (8).

In a variety of other behavioral health fields, digital health interventions utilizing smartphone technology have found success (9). In particular, conversational agents (CAs) have been applied to a variety of chronic disease contexts to help coach individuals and offer behavioral lifestyle interventions (10–12). Such applications have been shown to build working alliances with users (13), leverage benefits of gamification (14), utilize techniques from psychotherapy (e.g., cognitive behavioral therapy, motivational interviewing) (15) and enhance behavioral coaching in a manner similar to human-delivered coaching (11, 12, 16–19). Importantly, these interventions can be designed in a low-cost and accessible manner (20), so they have high potential to scale widely and offer a healthcare service to those whom may be lacking in treatment coverage (21, 22).

In this paper, we overview a digital health intervention that leverages findings from digital health research, combining varied theoretical and treatment approaches from different health domains into a single lifestyle intervention. The smartphone app, Elena+, named in honor of the Italian Nurse Elena Pagliarini, who was photographed exhausted from treating COVID-19 patients, has been developed within a short-time frame by a team of researchers from around the world. It aims to capitalize upon the key findings from behavioral and digital health fields to implement current best practices for the public good during the current emergency in public health. The Elena+ app, freely available on both iOS and Android devices in a variety of nations (United Kingdom, United States, Switzerland, Ireland, Spain, Colombia, Mexico), utilizes a CA to interact with users and offers personalized coaching in lifestyle health topics which may be under strain during this COVID-19 period. Topics included are: (i) COVID-19 health information, (ii) physical activity, (iii) mental health (loneliness, anxiety, utilizing mental resources), (iv) sleep and (v) diet and nutrition. The project also lays the groundwork for future interventions by providing open-source intervention logic, content, and software, which may serve as a useful start-point for tackling other chronic and mental illness and/or provide the basis of a digital control condition for other digital health interventions.

In addition to its function as a publicly available coaching tool Elena+ also doubles as a single-arm interventional study where we track individual progress through a series of psychoeducation and activity-based coaching sessions. The research aims of Elena+ are as follows; first, we measure changes from baseline to follow-up health assessments for each topic; second, we track self-reported behavioral intentions and actual behaviors during the coaching progress, to see if these measures mediate the change process, as demonstrated by differences over time in a topic's main assessment health outcome as outlined above; third, we capture data related to the patient profile and usage experience, to understand how the success of Elena+ may vary based on clusters of user/usage characteristics, that will enable better segmenting of the population and tailoring of approaches in future health interventions (23–25).

## Intervention Design

Elena+ is a smartphone app that uses a CA to lead individuals through a series of psychoeducation coaching sessions, comprising primarily of psychoeducational materials, behavior change activities, planning activities and intention/goal formation. Created rapidly to meet the emergent COVID-19 coronavirus pandemic, Elena+ delivers coaching sessions created by experts in their respective fields to attempt to reach in-need/at-risk sub-populations. A variety of intervention components have been utilized to: (i) foster engagement with the Elena+ app, and (ii) boost care potential in delivering pandemic lifestyle health outcomes. This harmonizes the intervention with current best-practice from digital service and health intervention fields as far as possible, whilst considering current time and resource constraints in developing the app. For the intervention design of Elena+, we combined various theory-driven and practice-led approaches from different fields and tailored coaching materials accordingly. In total 43 coaching sessions have been created on the topics of: COVID-19 health information, physical activity, loneliness, anxiety, utilizing mental resources, sleep, and diet/nutrition. A full overview of coaching topics and subtopics within them is given in section Coaching Topics.

**Figure 1:**
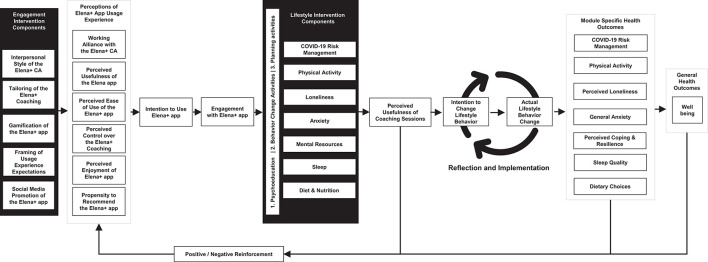
Elena+ conceptual model.

The conceptual model in Figure 1 overviews the driving engine of the Elena+ intervention, outlining how intervention components target: (i) theoretical constructs, (ii) antecedent causes of behavioral intentions, and (iii) behavioral activation. The Elena+ Engagement Intervention Components outline design choices for the app usage experience, the CA, and the promotional strategy aimed to promote positive perceptions of the Elena+ app usage experience. As stated in both Theory of Planned Behavior (TPB) (26) and Technology Acceptance Model (TAM) (27), once sufficiently positive evaluations of the app usage experience are created, users will intend to use the app, and thus exhibit a state of engagement. Once engaged, individuals are then able to benefit from the Lifestyle Intervention Components of: (i) psychoeducation, (ii) behavior change activities, and (ii) planning activities in the respective seven coaching topics. This in turn influences the perceived usefulness of the coaching sessions, and by completing further psychoeducational coaching content and activities, individuals will exhibit greater self-efficacy and feelings of social support (28). This empowers coachees to set behavioral intentions and follow them through as “actual behaviors” in their daily lives, during a period of reflection, implementation, and experiential learning as part of the coaching process (29). As individuals apply psychoeducation and behavior change/planning activities into their own lives, health outcome assessment scores are expected to improve leading to positive reinforcement loop via greater engagement with the Elena+ app. The intervention is therefore unique in its broad approach to target multiple facets of an individual's lifestyle, and varies from other CA interventions which typically focus on a single health domain but in greater depth (10) representing both a novel treatment and research opportunity in digital public health efforts (30).

### Intervention Components

#### Engagement Intervention Components

##### Interpersonal Style of the Elena+ CA

The working alliance represents the relationship quality between patients and healthcare professionals, and is robustly linked to treatment success in both offline and digital settings (13, 31–33). Comprising of task, bonds and goals (34) shared between coach and coachee, it is a key predictor of health behavior and attitude change (35). In digital contexts it can be boosted by creating interactions that adhere to principles outlined in positive psychology coaching and motivational interviewing (18, 35–37) such as leveraging interpersonal cues (38), expressing empathy (39), and eliciting change talk (29, 37, 40, 41). For Elena+ we utilize past findings such as blending both social and task-oriented dialogue (42), depiction of a pictorial avatar representation for the agent (38, 43, 44), and utilization of some backstory for the CA (45, 46) (i.e., it is a digital representation of a real nurse like Elena that helps fight COVID-19). In this vein, to help nurture a coaching atmosphere, terms such as “menu” or “next time you use the app” are avoided, and terms relevant to face-to-face communication such as “coaching choices” or “in your next coaching session” are utilized by the CA.

##### Personalization of Elena+ Coaching

Personalization is a key factor eliciting trust, feelings of familiarity and adoption of technologies (47) and in turn positively influences the user experience (48). Efforts were therefore put into giving user choice and autonomy as far as possible throughout the experience, and using non-forceful language in line with self-determination theory and motivational interviewing (49–51). Examples include the selection of your digital coach (Elena or Elliot), use of an assessment quiz to make topic recommendations, and the opportunity to set personalized goals related to behavior change. Additionally, Elena+ also offers “on demand coaching” i.e., individuals may continue coaching at any time they wish, and there are no limits on content availability which differs from other digital health interventions which limit content available per day.

##### Gamification

Gamification i.e., “gaming elements used outside of games” is a key factor in motivating users (52) and creating an engaged state (14, 53). It has been successfully used in mental health and psychiatry care (52) by applying gaming mechanics to non-game contexts (45), for example, winning points to add instrumental and experiential value to activities (54).

For the current Elena+ intervention, we utilize gamification concepts by rewarding individuals with badges to symbolize progress through the app, and awarding hearts for coaching session/assessment question completion, helping to evoke behavioral economic aspects related to avoiding losses and maximizing gains (55). Surprise bonus hearts are also awarded during the intervention, which has been found to instill additional motivation via random variable scheduling of rewards (56). These bonuses may be awarded for timely completion of all intervention content (whereby timely consists of completing 1–2 coaching sessions per day, requiring ~70 days to complete). This serves as a control mechanism to nudge coachees to complete Elena+ in an optimal time frame so that users' can adequately process and apply coaching content to their daily lives (see section Intervention Timeframe). Additionally, hearts are framed to coachees with a social message during the intervention onboarding; “hearts help our healthcare heroes,” with further details explaining to coachees that by earning hearts, individuals are following a path of action that helps frontline carers safe by ensuring healthcare systems are not overloaded. This is to strengthen altruistic and social motivation for using the app, which has been linked to internalized values and intrinsic forms of motivation (57, 58).

##### Framing of Usage Experience Expectations

Positively framing user expectations on usage experience enables feelings of transparency and value co-creation (59), and has been linked to increased behavioral intentions (60), relationship formation (61), and satisfaction (62). In Elena+ “onboarding” disclosures (i.e., disclosures from the CA related to service experience) are utilized during the first interactions and prior to selection of coaching topics for the first time a topic category is chosen, orienting coachees to the coaching process. Additionally, research has shown that failure to disclose privacy information in a transparent way may cause individuals to drop-out from digital services and cause poor trust in platforms (41). A recent review of CA use in digital health interventions has recommended disclosing privacy information transparently to avoid such complications (13). In this spirit, the Elena+ CA briefly outlines how information is kept safe and stored in an anonymous fashion within the chat, in addition to the minimum legal requirements of displaying the terms and conditions.

##### Social Media Promotion of the Elena+ App

Social media has been harnessed both as a tool to recruit participants (63) as well as for health promotion and behavior change (64). For the current intervention we created social media accounts on Facebook, Twitter, Instagram, and LinkedIn, as well as a separate website. At present the Facebook Ad Manager platform is used actively for both promotional and recruitment purposes through use of advertisements. As the project evolves, we may experiment with use of other platforms.

#### Lifestyle Intervention Components

##### Psychoeducation

Health literacy has been defined as “the cognitive and social skills which determine motivation and ability of individuals to gain access to, understand and use information in ways which promote and maintain good health” (65). On the population level, promoting patient health literacy is linked to reduced chronic illness prevalence, reduced early mortality and effective use of health services (66), while on a patient level, it represents the “personal and relational factors that affect a person's ability to acquire, understand and use information about health and health services” (67).

In Elena+ psychoeducation is employed “to enhance the likelihood of provision and receipt of effective and collaborative health care” (66) and boost patients' health literacy. As the app is aimed broadly at the general public, several steps have been taken to better ensure the understandability of psychoeducational material (68). These include; writing health information using layman's terms where possible, using specific terms with definitions, and allowing individuals the option to enquire for further explanations and definitions, whilst also ensuring the coaching experience is not unnecessarily slowed for high literacy coachees (69). An additional consideration was the division of materials across the intervention into beginner and intermediate levels (where appropriate) to better match individual knowledge, experiential background and/or motivational state (as detailed in section Coaching Topics).

##### Behavior Change Activities

To support the psychoeducation efforts that influence behavioral intention formation (70, 71), behavior change activities are utilized as part of the coaching and change process (29, 72). These activities are taken from a variety of specific fields (motivational interviewing, cognitive behavioral therapy) and adapted to the digital CA coaching context (18). Examples of activities used include the “5 good things technique,” cognitive restructuring, mindfulness/breathing exercises, anxiety diaries and more (40, 73, 74) depending on the coaching topic covered (see section Coaching Topics). This helps bring psychoeducational material to light by outlining practical techniques individuals can utilize to manage their health in the short term, as well as teaching longer term coping skills to manage symptoms in new contexts.

##### Planning Activities

Behavioral supports such as planning activities can be effective in simplifying decision making across the patient health literacy spectrum, helping to set clear and specific goals (75). They are particularly important for low health literacy groups whom may struggle in comprehending and applying information to their own lifestyles without support (76). In Elena+ planning activities are utilized to aid goal formation, for example, in the physical activity module; goal setting outcomes, goal setting behaviors, discrepancy between current behavior and idealized outcomes are discussed. Individuals can also set and review physical activity goals in line with the Capability, Opportunity, Motivation, Behavior (COM-B) approach (77). Additionally, at the end of each session, individuals are encouraged to set behavioral intention(s) based on content covered, offering concrete next steps to facilitate their behavioral activation (78). As the danger of the “intention-behavior” gap exists (79–81), individuals' actual behaviors are followed up upon *via* messaging 4–10 days following completion of a coaching session. This helps sync with extant research on using situational cues (82), and short text-message reminders of action-plans (83), to increase the effectiveness of goal/behavior planning activities.

### Coaching Topics

In the Elena+, individuals may complete coaching content in several different health topics. These represent the pandemic lifestyle areas where aforementioned techniques detailed in the intervention components (such as psychoeducation or planning activities) are applied to each health domain. For any given overarching coaching topic (e.g., Anxiety), a variety of sub-topics (e.g., “Breathing Away Anxiety”) are available for completion with the CA, each lasting ~5–10 min (see Table 1). Coaching for each subtopic content was created by an international and interdisciplinary team of domain health experts during April and May 2020, based on scientific findings and their expertise, and is continually reviewed regarding any new COVID-19 guidance from the World Health Organization (WHO).

**Table 1:** Coaching topic overview.

**Module topic**	**Beginner coaching subtopics**	**Intermediate+ coaching subtopics**
COVID-19	What is COVID-19 and what are coronaviruses?	What are pandemics and why do they occur?
	What are the symptoms and how do they differ from the flu?	How and when should I self-isolate?
	How is COVID-19 coronavirus spread?	How can I get tested/diagnosed for COVID-19?
	What groups are most at risk?	Are hospitals/medical facilities safe to visit?
	How can we prevent the spread?	More advanced information on preventing transmission/catching COVID-19
Physical activity	What is physical activity and how much should I do?	How does physical activity affect my immune system?
	What are the benefits of being active?	Safety, inspiration and fitness goals during COVID-19
	Getting more active during COVID-19	How can I improve my fitness?
	Safe exercising during COVID-19	How can I maximize the benefits of physical activity?
Sleep	Why is sleep important?	What is sleep hygiene?
	How does healthy sleep help to protect me from COVID-19?	What hinders and helps good sleep?
	Is good sleep important for my mental health?	How does poor sleep put me at risk for COVID-19?
	What happens if I do not sleep well?	How can I manage to sleep well during confinement?
	Can anxiety, stress and poor sleep cause COVID-19?	
Diet and nutrition	Unhealthy food hazards	
	The positive effects of a nutrition-rich diet	
	Preparing meals with the daily dozen	
Anxiety	What is anxiety and why is it hard to control?	
	COVID-19, risk perception and anxiety	
	How can I control my anxiety?	
	Breathing away anxiety	
	Confinement and anxiety	
Loneliness	What is loneliness?	
	Can loneliness make you sick?	
	How can we deal with loneliness?	
Mental resources	The fundamentals of mental resources	
	The functions of mental resources	
	The neuroscience behind mental resources	
	Identifying our mental resources	
	Activating our mental resources	

For the design of coaching content, we followed relevant theoretical approaches. For the topics of COVID-19 health information, sleep, diet and nutrition, and physical activity a Health Action Process Approach (HAPA) inspired design was used and we divided materials between beginner and intermediate+ difficulty levels so that Elena+ users with a greater degree of knowledge, experiential background and/or motivation (as discerned during a gamified health assessment, detailed in section Intervention Logic) are directed ahead to intermediate+ coaching materials to better promote their learning and engagement (84, 85). Presently, diet and nutrition contains three beginner coaching sessions with further intermediate+ materials planned. For the mental health topics (anxiety, loneliness, utilizing mental resources) intervention materials were conceptualized along a series of continua (rather than divided into discrete categories of beginner and intermediate+) following evidence-based transdiagnostic treatment in mental health (86–88). Lastly, in planning all topics, we also took inspiration from the Behavioral Change Wheel and COM-B model of behavior change (77), for example using the APEASE criteria to identify intervention functions and behavior change techniques suitable to deliver the COM-B components of psychological capability, reflective motivation, automatic motivation, and social opportunity (77). An overview of the rationale for including each coaching topic and specific content is given in the current section.

#### COVID-19 Health Information

Having individuals within society enact COVID-19 guidelines widely is vital for tackling the virus (8), however this requires a large information processing burden on the part of the individual, whom must vigilantly pay attention to developments. Additionally, one-way media channels must compete with a more interactive social media platforms, which presently contains the danger of misinformation and “fake news” (8), for example, stories claiming 5 g mobile-phone networks caused the COVID-19 outbreak (89). Certain individuals in traditionally at-risk groups lower in self-efficacy and/or health-literacy may be particularly vulnerable for a failure to follow guidelines (6, 90) and more susceptible to “fake news” stories (91). To tackle these factors, Elena+ offers the COVID-19 health information module based on trustworthy and legitimate sources including the core guidelines and information provided by the World Health Organization and other governmental bodies and/or charities/agencies.

#### Physical Activity

Current restrictions on individuals' lifestyles (gym closures, requirements to stay indoors, social distancing guidelines) are making physical activity levels more challenging to maintain (92). This may pose a particular issue for individuals that prefer socially oriented exercise contents (93, 94) (i.e., exercise classes, running with friends) and now experience a lack of motivation or structured guidance for solo exercise. The physical activity (PA) module delivers a variety of sub-topics and enables the development (or continuation) of physical activity routines to contribute to strengthened immune systems and improved population health. By providing information on suitable activity types (aerobic, strength, mixed) that individuals can perform whilst maintaining social distancing guidelines, we influence individual outcome expectancies, self-efficacy and goal setting to facilitate behavioral activation, and stay active while adhering to public health guidelines.

#### Sleep

Sleep is known to contribute to overall immune system health (95) and bolster mental health resilience (96), however, the pandemic creates additional stressors (such as increased use of electronic devices, less time spent physical active, additional stress) which has deleterious effects on sleep quality (97, 98). The sleep module therefore employs multicomponent cognitive behavioral therapy as recommended by the Standards of Practice Committee (SPC) of the American Academy of Sleep Medicine (AASM) (99) as an effective, non-pharmacological intervention to improve sleep hygiene (100, 101). Sleep education in Elena+ includes: (i) information about the neurophysiological components of sleep from the vigilance model (102), (ii) the different stages of sleep and associated oscillatory patters, (iii) the role of certain neurotransmitters (e.g., serotonin, dopamine) which help with the transition of said stages (102), as well as, (iv) explaining how sleep is regulated by the circadian rhythm (Process C) and the sleep-wake homeostasis (Process S) from the two-process model (103). Other sleep-relevant recommendations such as improving physical activity, nutrition, creating a sleep routine or reducing device use prior to bed are also discussed (97, 100, 104).

#### Diet and Nutrition

Diet and nutrition is known to contribute to the immune system and physical health generally (105). In the current period of COVID-19, ensuring individuals eat well and maintain a nutritional diet is key in boosting overall population health (106). Eating habits often become routine (107), and for many individuals their current eating routines will be in a period of flux; additional time at home may provide the chance to re-assess previous eating habits, and with sufficient guidance, allow individuals to develop new routines and exerting greater control over dietary choices. The module therefore functions to provide nutrition and diet information and support individuals in making healthy choices; influencing outcomes expectancies and self-efficacy regarding food and diet choices based upon guidelines from the World Health Organization (108) and publications recommending plant-centered diets in managing and preventing chronic diseases (109–111).

#### Mental Health

During the pandemic strains on mental health have been exhibited; with individuals experiencing periods of increased isolation, uncertainty regarding their employment and safety, and less freedom to enjoy stress-reducing activities (112, 113). Without guidance therefore, there exists the ever present danger that such stressors may lead to unhealthy coping strategies, creating a negative feedback loop and increased strains upon mental health (112). While mental health has previously been considered a delicate topic for automated agents to address, there exists a growing literature body on utilizing CAs to deliver high quality care (15, 19, 114). Contributing to this emergent stream of research, the mental health module offers the topics of: (i) anxiety, (ii) loneliness and (iii) discovering mental resources.

##### Anxiety

A CBT-inspired approach was used for anxiety materials, focusing on dysfunctional thoughts that affect behavior and functioning and emotion-regulation (115–117), whereby emotion regulation refers to decreasing the experiential and behavioral aspects of negative emotions (73). Some emotional regulation strategies utilized in Elena+ involve: (i) situation election (e.g., choosing not to watch or read news about COVID-19 throughout the day, only once or twice daily), (ii) attentional deployment (e.g., scheduling and directing attention to enjoyable activities activities), (iii) cognitive change (e.g., modifying how one appraises a situation so as to alter its emotional significance), and (iv) response modulation (e.g., using deep-breathing relaxation techniques) (73). In this way, the anxiety module has been developed considering the strategies recommended by Sanderson et al. (118), offering an evidence-based treatment for anxiety tailored toward pandemic circumstances.

##### Loneliness

Loneliness has been defined as the discrepancy between an individual's preferred and actual social relations (119). Prior to COVID-19 outbreak, loneliness was a major public health concern and had been linked to increased morbidity and mortality risks (120–122). As typical treatment for alleviating loneliness such as increasing opportunities for social interaction (123) is not presently possible, these issues are addressed by our intervention in several steps: (i) by discussing opportunities for social interaction compatible with COVID-19 recommendations, (ii) by implementing psychoeducation to make users aware of the increased risk of feeling lonely and its negative impact on well-being (124), and (iii) directly addressing these consequences in behavior change activities such pleasant activity scheduling (125).

##### Mental Resources

Coping resources have been found to reduce psychological distress and buffer the consequences of stressful life events (126, 127) however if individuals underestimate their potential to cope with stress, no adaptive coping strategies will be developed. Emotion-focused coping in Elena+ therefore adjusts an individual's emotional response to the varied and subjective stressors affecting them, and focuses on regulating negative emotional reactions to these stressors, following the Transactional Model of Stress and Coping by Lazarus and Folkman (128). This is done by using various cognitive and behavioral skills, such as positive-thinking, reframing, or distraction (e.g., defining a resource to enjoy such as writing down positive memories, utilizing relaxing space in the home or garden) with downstream consequences of boosting an individual's perceived competence and self-efficacy to manage stressors (129).

## Technical Implementation

### Intervention Logic

**Figure 2:**
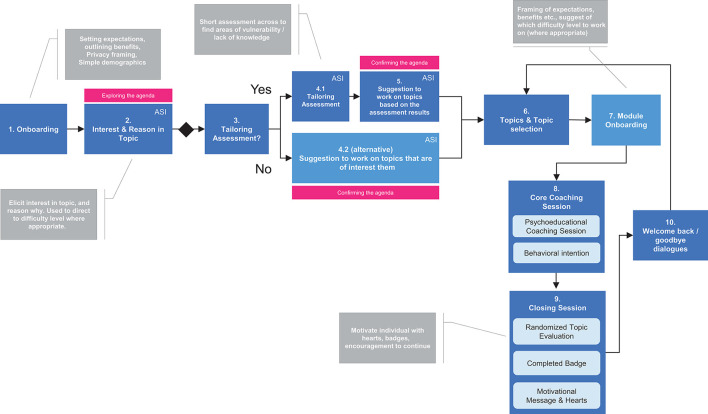
First use of Elena+. ASI, Agenda Setting Input.

**Figure 3:**
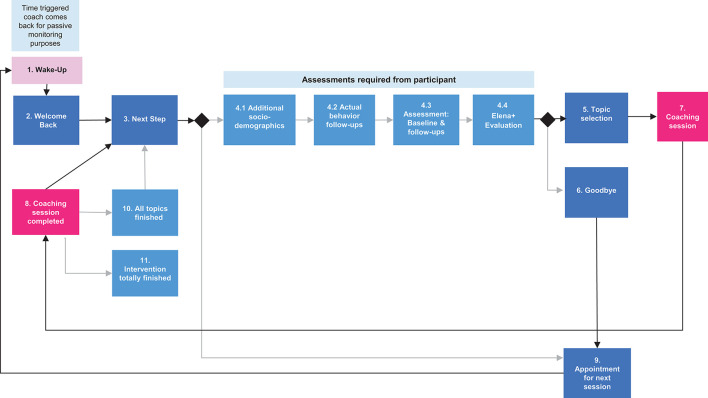
Ongoing use of Elena+.

The intervention logic for Elena+ is displayed in Figures 2, 3. Figure 2 shows the coachee process from download of the Elena+ app to completing their first coaching session.

Referring to Figure 2 (and to the diagram numbers), the intervention starts off by “*exploring the coaching agenda”* whereby: (1) coachees are onboarded to the Elena+ experience (i.e., explanations of the coaching service are given and expectations framed, privacy protection steps are mentioned) and simple demographics are taken, and (2) the coaching agenda is explored in greater depth where coachees input their coaching interest in the various coaching topics and optionally select from reasons why (e.g., the user states they are curious, or they have been struggling with a given topic), which is used as an input to tailor coaching content later between both topics and difficulty where appropriate. During the “*confirming the agenda”* stage (3–5) individuals are strongly encouraged to take the tailoring assessment which functions as a type of gamified quiz (4.1) by outlining benefits (i.e., the app will be better tailored to them and their circumstances). Based on the assessment results from this quiz, individuals are given a suggestion of which coaching topics are likely to be of most use to them and then they go to topic selection. Individuals may also opt to skip the assessment (4.2) to preserve user autonomy and go straight to topic selection (6). Following topic selection (6), individuals are directed to the module onboarding (7) which gives an overview of the benefits of taking this series of coaching topics and baseline assessment measures are taken, so that individuals pre and post health outcome scores from using coaching materials can be assessed at a later time. The module onboarding also occurs only once, on the first-time starting a given module.

A topic is then completed by an individual (8) which contains psychoeducation and behavior change/planning activities as appropriate, and a behavioral intention may be set based on the materials covered in that session. Typically, a single coaching session lasts from between 5 and 10 min. After this, individuals move to (9) closing session, where a badge is awarded for specific topic completion, hearts are also awarded for progress in the Elena+ coaching experience and a randomized session evaluation may occur (asking individuals how they perceived the coaching session). Lastly, individuals move to the (10) welcome back/goodbye dialogue where individuals can choose to: (i) continue coaching with another topic selection, (ii) set a date for the next coaching session, (iii) choose to “wake up” the coach to continue coaching at a non-scheduled time (e.g., before their next appointment).

Figure 3 shows ongoing use, whereby, individuals begin at the (2) “welcome back” dialogue (which can be coach or coachee triggered) and following this typically proceed to the next step (3) which may state that some questions (assessments) are required from the participant. If needed, the participant completes the (4) assessments, and typically proceeds to (5) topic selection and then (7) completes a coaching session, and when finished continues to (8) the closing session dialogue, subsequently choosing (3) next steps they would like to take. However, following the assessments, individuals may choose to also (6) finish, which may typically be the case when an individual has just completed a coaching session and does not wish to start another. This may also occur when an individual has completed all coaching sessions but continues to use Elena+ for behavioral reminders or to earn hearts to combat the pandemic (i.e., “hearts help our healthcare heroes” – as highlighted in Table 2). In either case, users would then go to (9) the “appointment for next session” dialogue (ANS) to set a date when Elena should come back to them for more coaching, and after this is confirmed a (1) wake up button is displayed where the user can trigger more coaching at any time. If the coachee does not select the wake-up button (1) then the coach will restart dialogue at (2) with the welcome back dialogue at the appointed time.

**Table 2:** Intervention components.

**Intervention components**	**Brief description**	**Theoretical background**
**Engagement intervention components**		
Interpersonal style of the Elena+ CA	Interpersonal and empathetic communication in line with coaching literature to increase relational between coach and coachee	Working Alliance, Horvath and Greenberg (130); Establishing and Maintaining Long-Term Human- Computer Relationships, Bickmore et al. (32).
Tailoring of the Elena+ coaching	Where possible, personalization is offered to tailor the intervention. Examples include the assessment quiz making tailored recommendations, self-selection of coaching topics, being available 24/7 for users between coaching session appointments. In line with coaching literature, individual choice and autonomy are preserved throughout, which also includes only making suggestions in a non-forceful manner.	Self-determination Theory, Ryan and Deci (49); Positive psychology coaching, Passmore and Oades (29)
Gamification of the Elena+ app	Gamification in the form of winning hearts and badges for demonstration of progress and motivational reinforcement	Serious Games and Gamification for Mental Health, Fleming et al. (52); Gamification for Health Promotion: Edwards et al. (131).
Framing of usage experience expectations	In line with services marketing, information systems research, and human-computer interaction research, we take inspiration in shaping first encounters through use of onboarding individuals regarding usage expectations and privacy matters.	Role Theory in the Service Encounter (132); The Onboarding Effect (133) Cardoso 2017; Communication Privacy Management Theory, Metzger (134)
Social media promotion of the Elena+ app	Advertisements and posts are used to promote, recruit and shape perceptions of the Elena+ app to adults 18+. At the time of writing, Facebook is actively used, we also have reserved Twitter, LinkedIn and Instagram accounts.	Using Social Media For Health Research, Arigo et al. (63); Harnessing Social Media for Health Promotion and Behavior Change, Korda and Itani (64)
**Lifestyle intervention components**		
Psychoeducation	Coaching sessions centered around health literacy information delivered by domain experts and put into an easy to understand format for those of varied health literacy levels.	Health Promotion, Nutbeam and Kickbusch (65), Health Literacy, World Health Organization (135).
Behavior change activities	Activities from certain coaching traditions as relevant to the domain (e.g. Cognitive Behavioral Therapy, Motivational Interviewing etc.) are used to put health information in context in the coachees life.	The Psychology of Coaching and Mentoring, Passmore et al. (37)
Planning activities	At the end of a coaching session, individuals are encouraged to set a behavioral intention. This synthesizes information participants may have learnt and by setting an intention, crystalizes it to a concrete next step, helping in the behavioral change process. Additionally, planning activities may be used during sessions with regard to straightforward plans to help implement behavioral intentions.	Health Action Process Approach, Schwarzer (85), Gollwitzer (136).

Lastly, the (10) “all topics finished” dialogue is triggered when an individual has completed all coaching topics, a congratulations message and additional hearts and summary of achievements are given. Individuals are given motivation for continuing to use the app (i.e., that it can keep you on track with intentions you have set) and a social motivation (answering assessment helps combat COVID-19). When all topics, assessments and actual behavior follow-ups are completed by individuals, the (11) “intervention totally finished” dialogue occurs, and the intervention ends for this participant.

### Development

Elena+ was developed during Summer 2020 using MobileCoach (www.mobile-coach.eu), an open source software platform, available under the industry and academic-friendly Apache 2 license (137) for smartphone-based and CA-delivered digital health interventions and ecological momentary assessments (138, 139). MobileCoach allows intervention authors to design fully automated data collection protocols and interventions consistent with the *talk-and-tools* paradigm (140). It offers a chat-based interface with free text/number input and predefined answer options that are used to simulate conversational turns commonly applied in counseling sessions with health professionals and their clients (the “talk”). The Elena app also delivers a wide range of “tools,” i.e., various micro-interventions such as reminders or psychoeducational video clips. Against this background, Elena+ can complement existing video mediated or personal counseling sessions with general practitioners, lifestyle coaches or mental health coaches, and can also reach individuals in a scalable way when a personal coaching approach is neither appropriate, nor geographically accessible, or beyond financial limits and personal resources (e.g., in epidemic times of isolation and social distancing). The intervention content for Elena+ is also available under the Creative Commons license CC BY-NC-SA, a non-commercial license that allows free access to content and requires the sharing of new developed features with the original intervention authors, to encourage scientific sharing and collaboration internationally. A screencast is available on the project website www.elena.plus as well as in the Supplementary Material.

## Evaluation of Elena+

### Sample and Data Collection

The Elena+ app is listed on both Apple/Google app stores as “Elena+ Care for COVID-19” (Spanish: “Elena+ cuidados ante la COVID-19”) and may be used on both iOS and Android devices in the United Kingdom, Ireland, Switzerland, and on Android devices only at present in the United States, Spain, Mexico, and Colombia with further launch countries planned. The app is listed in the search index with the following keywords (and Spanish equivalents): COVID-19, coronavirus, mental health, sleep, exercise, diet, nutrition, coaching, and thus a natural amount of organic recruitment occurs by individuals searching for these terms. In addition, we utilize the Facebook Ad Manager platform to target adults aged 18 or above, with no upper limits on age. At present we have capacity for up to two thousand users on our server and are currently exploring how to expand server capacity, so that more active users can be accommodated. Data collection began in June 2020.

Full ethical clearance was given by the ethical board of the university, ETH Zurich, for the project (application number: EK 2020-N-49) and the app content was reviewed by both Apple and Google who requested a list of scientific references to be included within the app before accepting. To use the app, users must: (i) be aged 18 years or over, (ii) accept the app terms and conditions and (iii) give informed consent for study purposes. If any of these are not true, individuals are screened out early in the Elena+ dialogue. All procedures were in accordance with the ethical standards of the 1964 Helsinki declaration and its later amendments.

Care was put into reducing unintended negative consequences. For example, during the tailoring assessment, individuals that score highly in the General Anxiety Disorder scale or Patient Health Questionnaire or (≥5) are recommended by the CA to seek human assistance (e.g., from their family doctor or a mental health charity) (141). As we gather no personally identifying information (as part of privacy protection measures) we cannot report these individuals who may benefit from human intervention to medical authorities nor compel individuals to seek out care. However, it was decided not to exclude these individuals from further use of the app, as while receiving human support would be optimal, receiving assistance through the Elena+ app is still better than receiving no support whatsoever. Regarding unintended consequences related to data safety, in the highly unlikely case of an attack on the Elena+ servers, user data will remain anonymous as we collect no personally identifying data, only simple non-identifiable information such as user gender, age, “nickname” and the language version in use. It could be possible that users specify their full name as their “nickname,” however, without further personally identifiable information collected (such as telephone number or e-mail address), it is extremely improbable individuals could be personally identified. Lastly, while efforts were put into making informed consent and app terms and conditions as understandable as possible (*via* dialogue from the CA in addition to the standard legal text displayed in all apps) it is possible that users could accept without fully understanding data will be used for analysis. Nonetheless, users always have the right to request the deletion of their data at any point in time.

### Study Design

Elena+ functions as a single-arm interventional study, whereby individuals' self-reported health assessment outcomes (see Table 3), the user-selected behavioral intentions at the end of each coaching session, and self-reported actual behaviors (see Table 4) are recorded as the core health and behavioral outcomes of interest. Additionally, we also collect ratings of the app usage experience, social demographic information and “marker variables” (i.e., user choices within the dialogues detailing the patient profile/usage experience objectively) shown in Table 5. Socio-demographic data is collected during first usage and before fifth subtopic completion, usage evaluations on a randomized basis after topic completion, and “marker” variables across all dialogues. Marker variables can be used to detail the user profile and usage experience, for example when a participant replies to coach that they do not understand terms used and need further explanation, this is saved as *health literacy marker variable*. Alternatively, if a user chooses to ask the CA for a joke, this choice is saved as a *humor marker variable*. As Elena+ has no control group, the authors plan to examine how individual attainment changes based on variations of app usage patterns and user profile over time.

**Table 3:** Baseline and follow-up assessments.

**For topic**	**Instrument**	**References**
COVID-19	COVID-19: risk perception and Coping strategies	Gerhold (142).
Diet and nutrition	Short survey instruments for children's diet and physical activity: the evidence	SAX Institute (143).
Sleep	Insomnia severity index: ISI-7	Bastien et al. (144).
Anxiety	General anxiety disorder: GAD-7	Spitzer et al. (145).
Loneliness	UCLA loneliness scale: ULS-6	Neto (146).
Physical activity	Single-item physical activity measure	Milton et al. (147)
	International physical activity questionnaire short form	YOUTHREX (148) and Booth (149)
Mental resources	Brief resilience coping scale	Sinclaire and Wallston (150).
Wellbeing	Patient health questionnaire: PHQ-2	Kronke et al. (151).

**Table 4:** Timing schedule of assessment and actual behavior questions in days.

**Topic**	**Actual behavior follow-ups:**				**Assessments:**					**±Days**
	**1st**	**2nd**	**3rd**	**4th**	**1st**	**2nd**	**3rd**	**4th**	**5th**	
Anxiety	7	21	35	77	14	28	42	84	126	0
Mental resources	8	22	36	78	15	29	43	85	127	+1
Loneliness	9	23	37	79	16	30	44	86	128	+2
Sleep	10	24	38	80	17	31	45	87	129	+3
Physical activity	6	20	34	76	13	27	41	83	125	−1
Diet and nutrition	5	19	33	75	12	26	40	82	124	−2
COVID-19info	4	18	32	75	11	25	39	81	123	−3

**Table 5:** Summary selection of marker variables.

**Marker variable**	**Explanation**
Literacy marker	Whether an individual is confused or not
Sedentary marker	Whether an individual is sedentary or not
Anxiety marker	Whether an individual is currently experiencing anxiety or not
Depression marker	Whether an individual is currently feels depressed or not
Weight marker	Whether an individual is reports struggling with their weight or not
Sleep Marker	Whether an individual is reports struggling with their sleep patterns or not
Humor marker	Whether an individual is engages in humor with the CA or not
More coaching marker	Whether an individual indicates desire for more coaching on a specific topic or not
Devices marker	Whether an individual uses many electronic devices or not
Loneliness marker	Whether an individual is currently feels lonely or not
Smoker marker	Whether an individual smokes tobacco or not

### Methods

To meet the project research aims, we will utilize panel data methods to track changes in health outcome scores gathered at baseline (i.e., after a topic has been selected but no coaching content has been completed yet) compared with follow-up intervals as individuals continue to use the app (timing specified in Table 4). To do this we will specify Auto-Regressive Moving Average (ARMA) models (152), whereby: (i) health outcome assessment scores are regressed on time, (ii) health outcome assessment scores are regressed on time, with no. of coaching subtopics completed specified as a moderating variable, (iii) health outcome assessment scores are regressed on time, with user selected behavioral intentions and user reported actual behaviors specified as serial mediators. A practical example of this would be examining whether setting intentions and reporting actual behaviors of abstaining from electronic devices before bed serially mediates the relationship between time spent using the app and lower scores for the Insomnia Severity Index.

Should sufficient observations be gathered, it may also be possible to adapt the aforementioned models and make forecasts based upon functions of past independent variable value(s) and/or past errors, as well as the present time error (153). In this case we would divide the dataset into training and test datasets, specify the ARMA model on training data, and compare its efficacy on test data. If suitable fit is found, the model may be used to make predictions (as to the impact of time spent using the app/no. of coaching subtopics completed/no. of intentions/actual behaviors reported) and their impact on health assessment scores. Additional analyses may include using socio-demographic and marker variables as inputs in Cluster Analyses such as Supervised k-Means Clustering (154) whereby individuals are grouped into sub-populations based upon health assessment scores and marker variables selected while using the app. In such a manner, it would be possible to identify aspects of the usage experience (for example, selection of humor marker variables, indicative of greater user engagement) that are linked to superior health outcome assessments.

### Intervention Timeframe

The Elena+ intervention timeframe is estimated to last for approximately half a year if individuals complete all intervention content, as well as all subsequent assessments and actual behavior monitoring questions. However, the time to complete all content fully depends upon how quickly individuals complete all coaching sessions, and the subsequent assessments and actual behavior questions that are triggered by completing coaching sessions. The schedule of follow up health assessments and actual behaviors questions is given in Table 4. We utilize some “intervention jitter” (i.e., variation in timing of follow-up questions after coaching sessions) so that assessments are less likely to co-occur on the same day and that the potential burden for users is lessened. For example, for the anxiety topic health outcome assessments occur from 14 days after the first anxiety coaching session, and actual behavior questions 7 days.

## Outlook

At the time of writing, Elena+ is available in three language versions: (i) English (in Switzerland, Ireland, the United Kingdom, and the United States), European Spanish (in Spain) and Latin American Spanish (in Mexico and Colombia) with data collection on going. Finished translations have been created for Tamil which will be launched in India, Sri Lanka, and Singapore. Work is also pending to improve server infrastructure which currently has capacity for 2,000 users.

Moving forward, increasing the effectiveness of Elena+ around the world may comprise of adapting and improving coaching content, features and visual aesthetics, for example, making cultural adaptations to match the profile of users in new launch countries. With the help of a sponsor, it could be possible to strengthen the social motivation for using Elena+ by having gamified hearts redeemable for certain medical equipment (for example, winning hearts providing a donation of money to supply personal protective equipment or ventilators to at-risk areas). In a similar vein, understanding the “state of receptivity” (155, 156) of participants and sending reminders and notifications at the correct moment may represent a fruitful research direction. Post-pandemic, Elena+ intervention materials may also be adapted to function as a type of digital control condition for other digital health interventions, which would help measure the degree of change attributed to the design choices of a specific digital treatment tailored to a certain disease content against a general lifestyle digital intervention. Researchers may then be able to assess the efficacy of their treatment, over and above the effect of simply using any type of digital tool.

Elena+ intervention content is available under the Creative Commons license 4.0 CC BY-NC-SA, and MobileCoach (the underlying software for Elena+) is available under the Apache 2 license. Materials have been made available to help foster a dynamic research community around Elena+, so that researchers can utilize, adapt, and build on our content, working autonomously in new contexts to add new features/contents whilst sharing findings with other intervention authors. To this end, a collaboration has begun with colleagues from Dartmouth College in the United States to adapt elements of Elena+ into a “just-in-time-adaptive intervention” (157) whereby sensor data (such as GPS location, date and time, Wi-Fi connection status) is utilized to match phone notifications to user state of receptivity (156). Additionally, discussions are underway with colleagues from Singapore-ETH Centre to build upon Elena+ dialogues for use in studies preventing type-2 diabetes and depression at the population-level. Further interested collaborators are warmly invited to contact the authors for further collaboration opportunities.

## Concluding Thoughts

Taken in total then, Elena+ represents a highly innovative digital health intervention developed at speed during the COVID-19 coronavirus pandemic to deliver *pandemic lifestyle care*. In the long term, Elena is not envisioned to be a static intervention, but rather an intervention which will evolve and adapt, leveraging the revolutionary potential of digital health to learn, innovate and apply solutions (30). Results will demonstrate areas of successes of the tool, as well as how improvements can be added to improve effectiveness of this and other future digital health interventions. These findings may be particularly applicable to other population-level threats to public health moving forward, such as obesity epidemic (158), and contribute to a greater understanding of digital health interventions for public health promotion.

## Ethics Statement

The studies involving human participants were reviewed and approved by ETH Zurich, Zurich, Switzerland. The patients/participants provided their written informed consent to participate in this study.

## Author Contributions

All authors listed have made a substantial, direct and intellectual contribution to the to the Elena+ project, and have approved the paper for publication.

## Funding

This research was supported by the National Research Foundation, Prime Minister's Office, Singapore under its Campus for Research Excellence and Technological Enterprise (CREATE) programme.

## Conflict of Interest

JO, PS, AA, DR, CB, OK, EF, FW, and TK are affiliated with the Centre for Digital Health Interventions www.c4dhi.org, a joint initiative of the Department of Management, Technology and Economics at ETH Zurich and the Institute of Technology Management at the University of St. Gallen, which was funded in part by the Swiss health insurer CSS Versicherung. EF and TK are also cofounders of Pathmate Technologies, a university spin-off company that creates and delivers digital clinical pathways. However, neither CSS nor Pathmate Technologies are involved in the Elena+ project or this article. The remaining authors declare that the research was conducted in the absence of any commercial or financial relationships that could be construed as a potential conflict of interest.

The reviewer AR declared a past co-authorship with several of the authors AS and RB to the handling editor.

## Publisher's Note

All claims expressed in this article are solely those of the authors and do not necessarily represent those of their affiliated organizations, or those of the publisher, the editors and the reviewers. Any product that may be evaluated in this article, or claim that may be made by its manufacturer, is not guaranteed or endorsed by the publisher.
