# Corrigendum: Elena+ Care for COVID-19, a Pandemic Lifestyle Care Intervention: Intervention Design and Study Protocol

**DOI:** 10.3389/fpubh.2021.809278

**Published:** 2021-11-26

**Authors:** Joseph Ollier, Simon Neff, Christine Dworschak, Arber Sejdiji, Prabhakaran Santhanam, Roman Keller, Grace Xiao, Alina Asisof, Dominik Rüegger, Caterina Bérubé, Lena Hilfiker Tomas, Joël Neff, Jiali Yao, Aishah Alattas, Veronica Varela-Mato, Amanda Pitkethly, Mª Dolores Vara, Rocío Herrero, Rosa Mª Baños, Carolina Parada, Rajashree Sundaram Agatheswaran, Victor Villalobos, Olivia Clare Keller, Wai Sze Chan, Varun Mishra, Nicholas Jacobson, Catherine Stanger, Xinming He, Viktor von Wyl, Steffi Weidt, Severin Haug, Michael Schaub, Birgit Kleim, Jürgen Barth, Claudia Witt, Urte Scholz, Elgar Fleisch, Florian von Wangenheim, Lorainne Tudor Car, Falk Müller-Riemenschneider, Sandra Hauser-Ulrich, Alejandra Núñez Asomoza, Alicia Salamanca-Sanabria, Jacqueline Louise Mair, Tobias Kowatsch

**Affiliations:** ^1^Centre for Digital Health Interventions, Department of Management, Technology and Economics, Eidgenössische Technische Hochschule (ETH) Zurich, Zurich, Switzerland; ^2^Department of Management, Technology, and Economics, Eidgenössische Technische Hochschule (ETH) Zurich, Zurich, Switzerland; ^3^Department of Psychology, University of Zurich, Zurich, Switzerland; ^4^Future Health Technologies, Singapore-ETH Centre, Campus for Research Excellence and Technological Enterprise (CREATE), Singapore, Singapore; ^5^School of Medicine, Johns Hopkins University, Baltimore, MD, United States; ^6^Executive School of Management, Technology and Law, University of St. Gallen, St. Gallen, Switzerland; ^7^School of Sport, Exercise and Health Sciences, Loughborough University, Loughborough, United Kingdom; ^8^Sport, Exercise and Health Sciences, Edinburgh Napier University, Edinburgh, United Kingdom; ^9^Polibienestar Research Institute, University of Valencia, Valencia, Spain; ^10^Centro de Investigación Biomédica en Red de Fisiopatología de la Obesidad y Nutrición (CIBERObn) Physiopathology of Obesity and Nutrition, Instituto de Salud Carlos III, Madrid, Spain; ^11^Department of Personality, Evaluation and Psychological Treatment, Faculty of Psychology, University of Valencia, Valencia, Spain; ^12^Department of Psychology, Universidad San Buenaventura, Bogotá, Colombia; ^13^National Institute of Education, Nanyang Technological University, Singapore, Singapore; ^14^Interdisciplinary Center for Health Workplaces, University of California, Berkeley, Berkeley, CA, United States; ^15^Centre for Digital Health Interventions, Institute of Technology Management, University of St. Gallen, St. Gallen, Switzerland; ^16^Department of Psychology, University of Hong Kong, Pokfulam, Hong Kong, SAR China; ^17^Department of Computer Science, Dartmouth College, Hanover, NH, United States; ^18^Center for Technology and Behavioral Health, Geisel School of Medicine, Hanover, NH, United States; ^19^Business School, Durham University, Durham, United Kingdom; ^20^Epidemiology, Biostatistics and Prevention Institute, University of Zurich, Zurich, Switzerland; ^21^Institute for Implementation Science in Health Care, University of Zurich, Zurich, Switzerland; ^22^Department of Psychiatry, Psychotherapy and Psychosomatics, University of Zurich, Zurich, Switzerland; ^23^Swiss Research Institute for Public Health and Addiction, University of Zurich, Zurich, Switzerland; ^24^Institute for Complementary and Integrative Medicine, University Hospital Zurich, University of Zurich, Zurich, Switzerland; ^25^Applied Social and Health Psychology, Department of Psychology, University of Zurich, Zurich, Switzerland; ^26^Dynamics of Healthy Aging, University of Zurich, Zurich, Switzerland; ^27^Family Medicine and Primary Care, Lee Kong Chian School of Medicine, Nanyang Technological University, Singapore, Singapore; ^28^Department of Medicine, Saw Swee Hock School of Public Health, Yong Loo Lin School of Medicine, National University of Singapore, Singapore, Singapore; ^29^Center for Digital Health, Berlin Institute of Health and Charité, Berlin, Germany; ^30^Department of Applied Psychology, University of Applied Sciences Zurich, Zurich, Switzerland; ^31^Unidad Académica de Cultura, Universidad Autónoma de Zacatecas, Zacatecas, Mexico

**Keywords:** chatbot, conversational agent (CA), digital coaching, digital health, coronavirus-COVID-19, gamification, mental health, pandemic lifestyle care

In the published article, there were errors regarding the affiliations of several authors.

For “Joseph Ollier”, instead of having affiliation “1,2”, they should have “1”. For “Olivia Clare Keller”, instead of having affiliations “1,2,15”, they should have “1,15”. For “Lorainne Tudor Car”, instead of having affiliations “3,27”, they should have “4,27”. For “Alicia Salamanca-Sanabria” instead of having affiliation “3”, they should have “4”. For “Jacqueline Louise Mair”, instead of having affiliation “3”, they should have “4”. For “Tobias Kowatsch”, instead of having affiliation(s) “1,2,15,28”, they should have “1,4,15”.

In the published article, there was also an error in affiliation “29”. Instead of “Center for Digital Health, Berlin Institute of Health and Charité, Berlin, Germany”, it should be “Center for Digital Health, Berlin Institute of Health at Charité, Berlin, Germany”.

There was also an error in affiliation “4”. Instead of “Future Health Technologies Programme, Singapore-Eidgenössische Technische Hochschule (ETH) Centre at Campus for Research Excellence and Technological Enterprise (CREATE), Singapore, Singapore”, it should be “Future Health Technologies, Singapore-ETH Centre, Campus for Research Excellence and Technological Enterprise (CREATE), Singapore”. Additionally, there was an error in affiliation “23” instead of “Swiss Research Institute for Public Health and Addiction, Zurich University, Zurich, Switzerland” it should be “Swiss Research Institute for Public Health and Addiction, University of Zurich, Zurich, Switzerland”.

The authors apologize for these errors and state that this does not change the scientific conclusions of the article in any way. The original article has been updated.

## Publisher's Note

All claims expressed in this article are solely those of the authors and do not necessarily represent those of their affiliated organizations, or those of the publisher, the editors and the reviewers. Any product that may be evaluated in this article, or claim that may be made by its manufacturer, is not guaranteed or endorsed by the publisher.

