# Supplementary material for: Elena+ Care for COVID-19, a Pandemic Lifestyle Care Intervention: Intervention Design and Study Protocol
Source: Front Public Health. 2021 Oct 21;9:625640. doi: 10.3389/fpubh.2021.625640 (PMC8566727; doi:10.3389/fpubh.2021.625640)
Supplement: Supplementary file 3 [file Data_Sheet_1.docx]

Supplementary Material

# Supplementary Figures and Tables

| Instrument | Reference |
| --- | --- |
| Net Promoter Score | Reichheld, 2003 [172] |
| Session Alliance Inventory | Falkenström et al, 2015 [173] |
| Session Behavioral Facilitation*“As a result of this session with $coachName, I am clearer as to how I can change my behaviors.”* | Self-created single item |
| Perceived Usefulness | Shih et al, 2019 [68]; Davis, 1989 [28] |
| Perceived Control | Shih et al, 2019 [68]; Davis, 1989 [28] |
| Perceived Enjoyment | Shih et al, 2019 [68]; Davis, 1989 [28] |
| Perceived Ease of Use | Shih et al, 2019 [68]; Davis, 1989 [28] |

**Supplementary Table 1**. Other Measures Captured

## Supplementary Figures


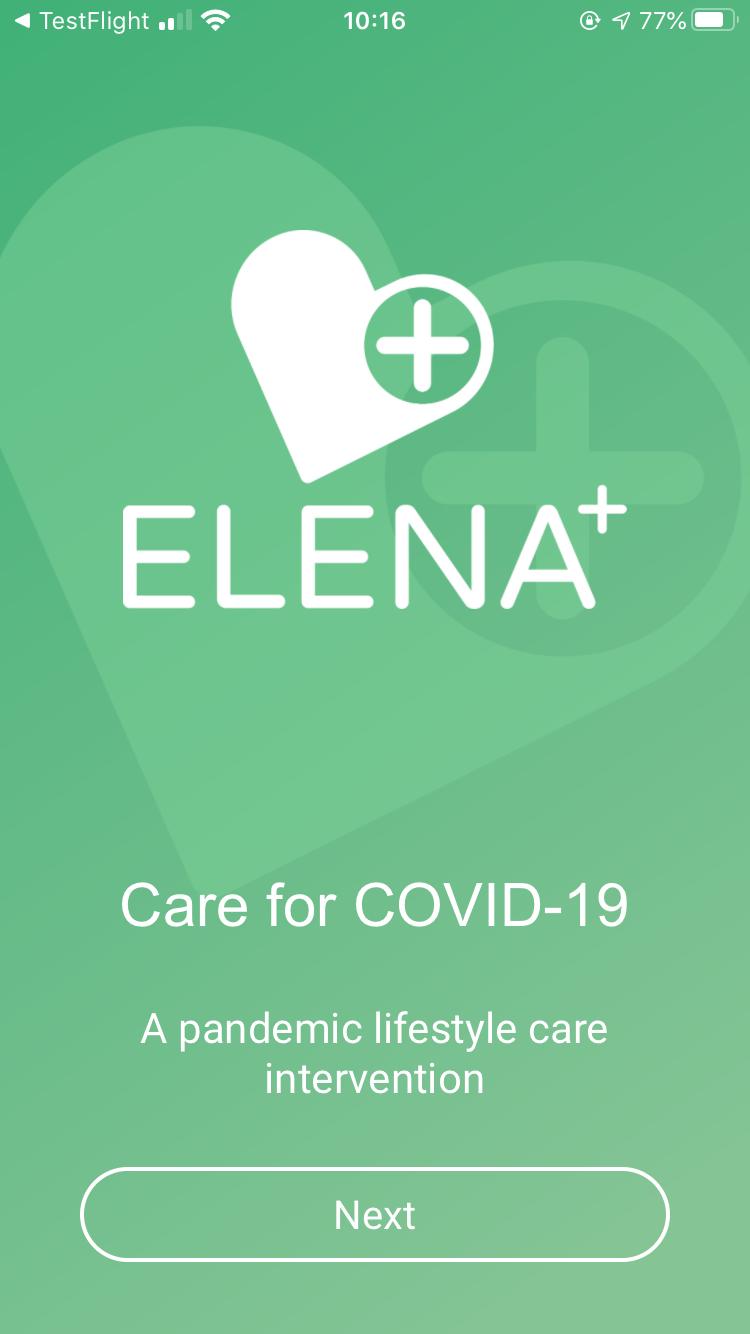

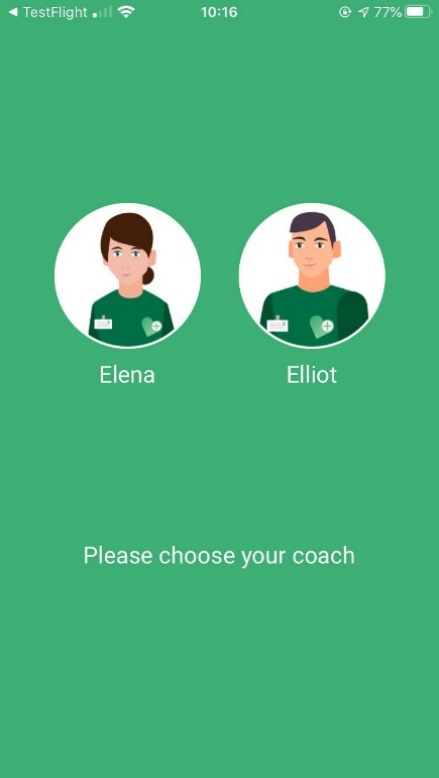


**Supplementary Figure 1.** Start screens of Elena+


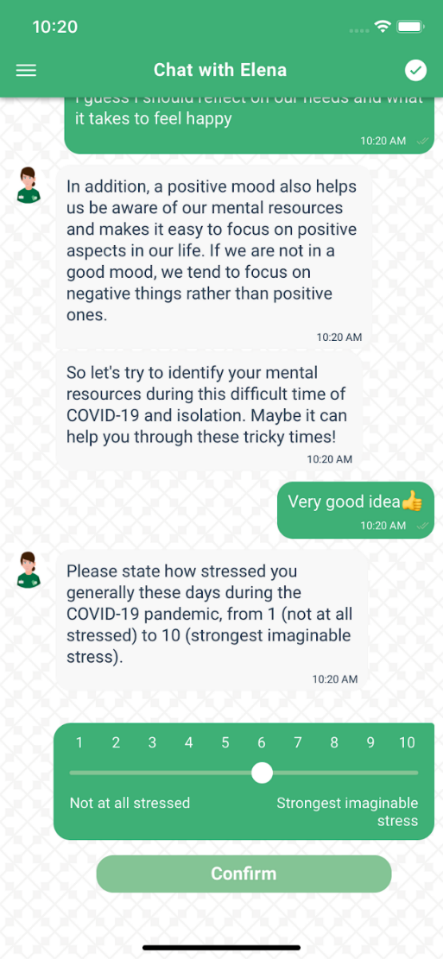

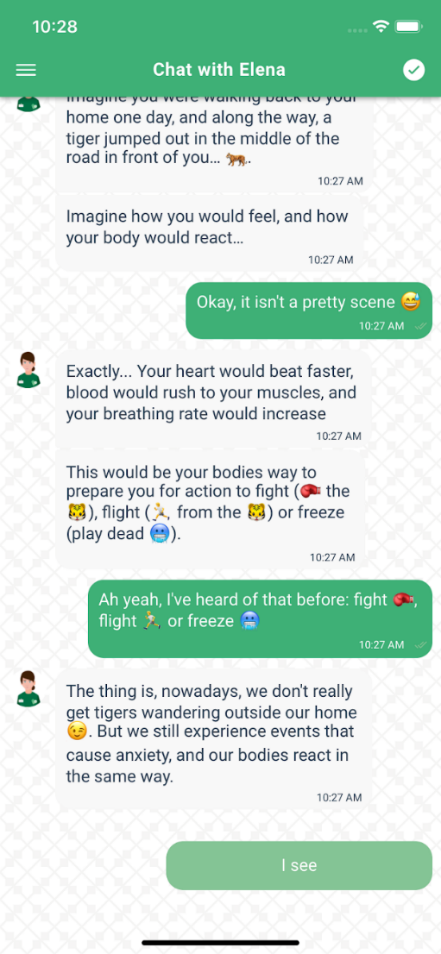


**Supplementary Figure 2.** Elena+ Dialogue

**
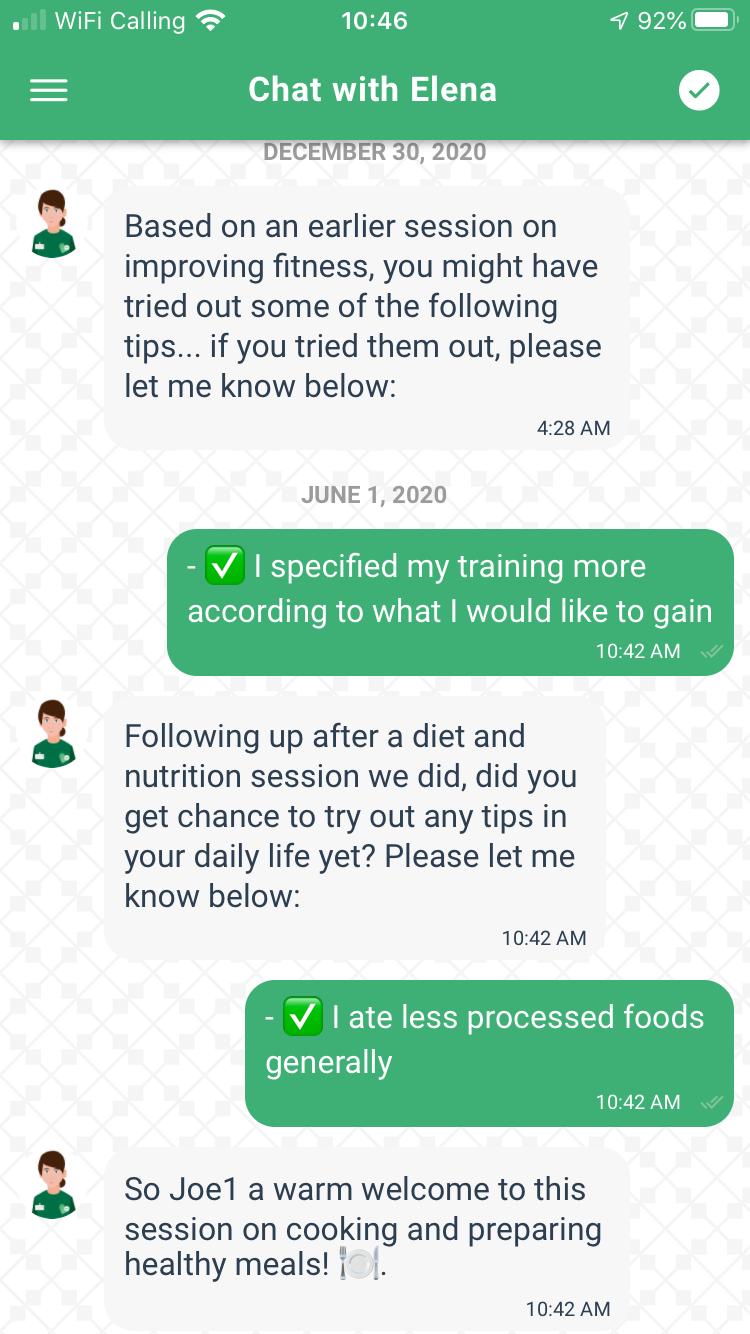

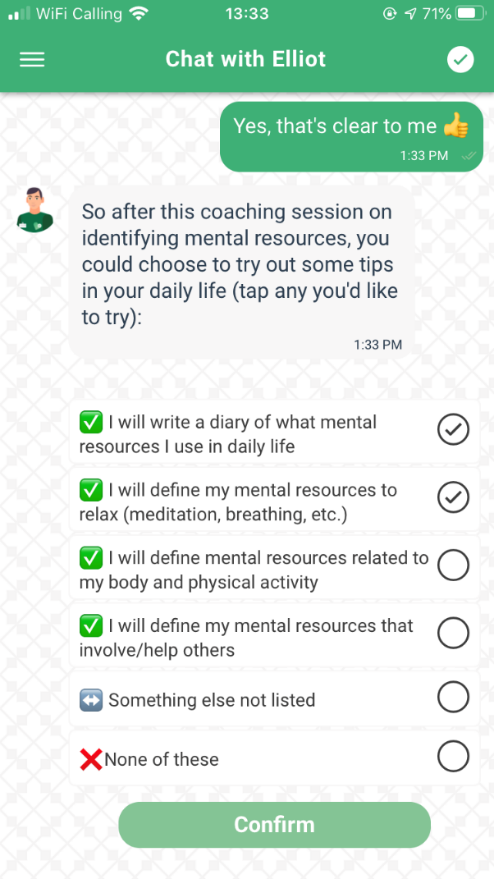
**

**Supplementary Figure 3.** Setting Intentions and Reporting Behaviors


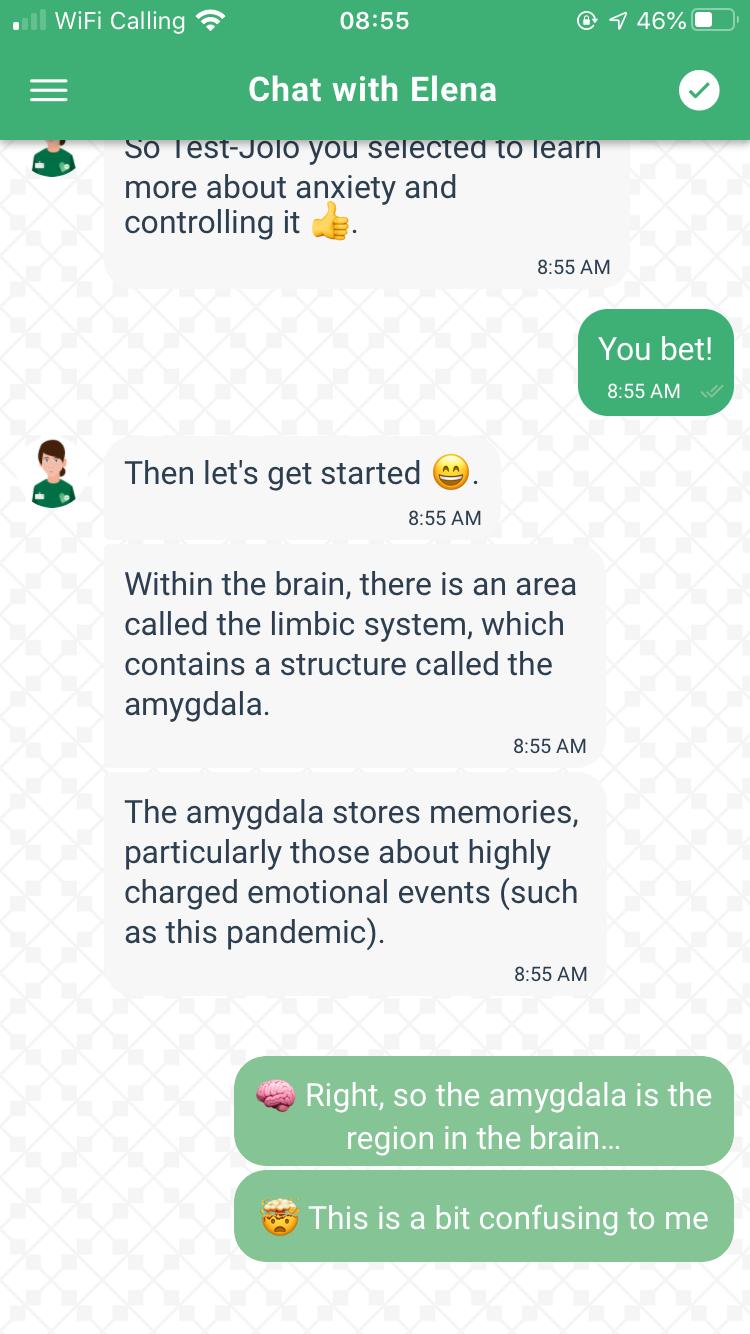

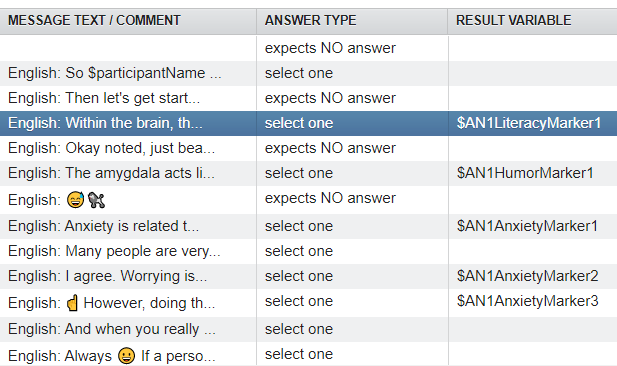


**Supplementary Figure 3.** Marker Variables in Dialogue and Mobile Coach Platform
